# 3-(1,3-Dithio­lan-2-yl­idene)-1-phenyl­pyridine-2,4(1*H*,3*H*)-dione

**DOI:** 10.1107/S1600536809021862

**Published:** 2009-06-17

**Authors:** Lei-Jiao Li, Yan Li, Xi-Yun Hao, Xiu-Yun Sun

**Affiliations:** a School of Chemical and Pharmaceutical Engineering, Jilin Institute of Chemical Technology, Jilin 132022, People’s Republic of China

## Abstract

The title compound, C_14_H_11_NO_2_S_2_, was synthesized by reaction of 2-(1,3-dithio­lan-2-yl­idene)-3-oxo-*N*-phenyl­butanamide with *N*,*N*′-dimethyl­formamide dimethyl acetal in *N*,*N*′-dimethyl­formamide. The mol­ecule exhibits a V-shaped conformation in the crystal, with a dihedral angle of 65.9 (2)° between the benzene and pyridine rings. In the crystal. C—H⋯O and C—H⋯S interactions are observed. Two C atoms of the dithiolane ring are disordered with occupancies in the ratio 0.541 (13)/0.459 (13).

## Related literature

For the synthesis, see Li *et al.*, (2008 ▶).
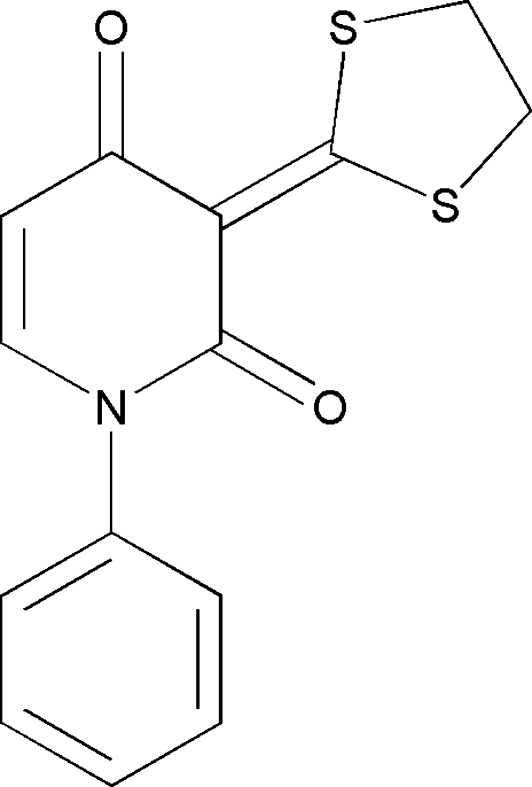


## Experimental

### 

#### Crystal data

C_14_H_11_NO_2_S_2_
*M* *_r_* = 289.36
Monoclinic, 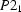

*a* = 5.7708 (17) Å
*b* = 12.033 (4) Å
*c* = 9.624 (3) Å
β = 101.094 (3)°
*V* = 655.8 (4) Å^3^
*Z* = 2
Mo *K*α radiation
μ = 0.40 mm^−1^
*T* = 292 K
0.35 × 0.29 × 0.28 mm

#### Data collection

Bruker APEXII CCD diffractometer
Absorption correction: multi-scan (*SADABS*; Bruker, 2002 ▶) *T*_min_ = 0.870, *T*_max_ = 0.894
5598 measured reflections
2543 independent reflections
2361 reflections with *I* > 2σ(*I*)
*R*_int_ = 0.050

#### Refinement

*R*[*F* ^2^ > 2σ(*F* ^2^)] = 0.029
*wR*(*F* ^2^) = 0.071
*S* = 1.04
2543 reflections
191 parameters
1 restraint
H-atom parameters constrained
Δρ_max_ = 0.17 e Å^−3^
Δρ_min_ = −0.21 e Å^−3^
Absolute structure: Flack (1983 ▶), 1181 Friedel pairs
Flack parameter: −0.05 (5)

### 

Data collection: *APEX2* (Bruker, 2007 ▶); cell refinement: *SAINT* (Bruker, 2007 ▶); data reduction: *SAINT*; program(s) used to solve structure: *SHELXS97* (Sheldrick, 2008 ▶); program(s) used to refine structure: *SHELXL97* (Sheldrick, 2008 ▶); molecular graphics: *SHELXTL* (Sheldrick, 2008 ▶); software used to prepare material for publication: *SHELXTL*.

## Supplementary Material

Crystal structure: contains datablocks global, I. DOI: 10.1107/S1600536809021862/rk2145sup1.cif

Structure factors: contains datablocks I. DOI: 10.1107/S1600536809021862/rk2145Isup2.hkl

Additional supplementary materials:  crystallographic information; 3D view; checkCIF report

## Figures and Tables

**Table 1:** Hydrogen-bond geometry (Å, °)

*D*—H⋯*A*	*D*—H	H⋯*A*	*D*⋯*A*	*D*—H⋯*A*
C1′—H1′*A*⋯O1^i^	0.97	2.43	3.300 (17)	148
C2—H2*B*⋯O1^i^	0.97	2.69	3.388 (8)	129
C7—H7⋯O2^ii^	0.93	2.36	3.259 (3)	163
C14—H14⋯O2^iii^	0.93	2.46	3.293 (3)	149
C11—H11⋯S1^iv^	0.93	2.90	3.756 (3)	153

Symmetry codes: (i) 
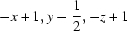
; (ii) 
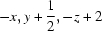
; (iii) 
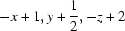
; (iv) 
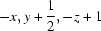
.

**Fig. 1.**
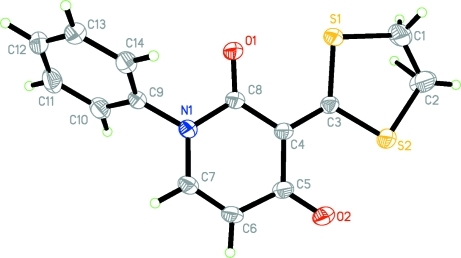
Molecular structure of the title compound with atom numbering scheme. Displacement ellipsoids are drawn at the 30% probability level. H atoms are presented as a small spheres of arbitrary radius. Only major fragment of disordered cycle is presented.

## supplementary crystallographic information

### Comment

*N*-Substituted pyridone compounds have versatile physiological activity. For instance, they can sterilize, ease pain, resist tumour and cure parkinsonism and so forth. These compounds can cure β-thalassemia beyond compare, beacuse they have powerful ability to form coordination compounds. In the molecule of the title compound, (Fig. 1), C1 and C2 are disordered in ratio 0.541 (13)/0.459 (13). The molecule exhibits a V-shaped conformation in the crystal with a dihedral angle of 65.9 (2)° between the benzene ring and the pyridine ring. The dihedral angle between the pyridine ring and the dithiolane ring is 2.6 (8)°.

### Experimental

The title compound, with M.P. 497 K, was synthesized according to the literature (Li *et al.*, 2008). It was dissolved in ethyl acetate at room temperature and hexane was added. The solution was kept at room temperature in a sealed flask for a few days to give single crystals suitable for single crystal X-ray analysis.

### Refinement

All H atoms bound to C atoms were generated geometrically and refined as riding atoms with C–H = 0.93Å for aromatic H and 0.97Å for CH_2_ groups, with *U*_iso_(H) = 1.2 *U*_eq_(C).

### Crystal data

**Table:**

C_14_H_11_NO_2_S_2_	*F*(000) = 300
*M**_r_* = 289.36	*D*_x_ = 1.465 Mg m^−^^3^
Monoclinic, *P*2_1_	Melting point: 497 K
Hall symbol: P 2yb	Mo *K*α radiation, λ = 0.71073 Å
*a* = 5.7708 (17) Å	Cell parameters from 3388 reflections
*b* = 12.033 (4) Å	θ = 2.7–26.1°
*c* = 9.624 (3) Å	µ = 0.40 mm^−^^1^
β = 101.094 (3)°	*T* = 292 K
*V* = 655.8 (4) Å^3^	Block, yellow
*Z* = 2	0.35 × 0.29 × 0.28 mm

### Data collection

**Table:**

Bruker APEXII CCD diffractometer	2543 independent reflections
Radiation source: fine-focus sealed tube	2361 reflections with *I* > 2σ(*I*)
graphite	*R*_int_ = 0.050
ω scans	θ_max_ = 26.1°, θ_min_ = 2.7°
Absorption correction: multi-scan (*SADABS*; Bruker, 2002)	*h* = −7→7
*T*_min_ = 0.870, *T*_max_ = 0.894	*k* = −14→14
5598 measured reflections	*l* = −11→11

### Refinement

**Table:**

Refinement on *F*^2^	Secondary atom site location: difference Fourier map
Least-squares matrix: full	Hydrogen site location: inferred from neighbouring sites
*R*[*F*^2^ > 2σ(*F*^2^)] = 0.029	H-atom parameters constrained
*wR*(*F*^2^) = 0.071	*w* = 1/[σ^2^(*F*_o_^2^) + (0.035*P*)^2^] where *P* = (*F*_o_^2^ + 2*F*_c_^2^)/3
*S* = 1.04	(Δ/σ)_max_ = 0.001
2543 reflections	Δρ_max_ = 0.17 e Å^−^^3^
191 parameters	Δρ_min_ = −0.21 e Å^−^^3^
1 restraint	Absolute structure: Flack (1983), 1181 Friedel pairs
Primary atom site location: structure-invariant direct methods	Flack parameter: −0.05 (5)

### Special details

**Table:**

Geometry. All s.u.'s (except the esd in the dihedral angle between two l.s. planes) are estimated using the full covariance matrix. The cell s.u.'s are taken into account individually in the estimation of s.u.'s in distances, angles and torsion angles; correlations between s.u.'s in cell parameters are only used when they are defined by crystal symmetry. An approximate (isotropic) treatment of cell s.u.'s is used for estimating s.u.'s involving l.s. planes.
Refinement. Refinement of *F*^2^ against ALL reflections. The weighted *R*-factor *wR* and goodness of fit S are based on *F*^2^, conventional *R*-factors *R* are based on *F*, with *F* set to zero for negative *F*^2^. The threshold expression of *F*^2^ > 2σ(*F*^2^) is used only for calculating *R*-factors(gt) etc. and is not relevant to the choice of reflections for refinement. *R*-factors based on *F*^2^ are statistically about twice as large as those based on *F*, and *R*-factors based on ALL data will be even larger.

### Fractional atomic coordinates and isotropic or equivalent isotropic displacement parameters (Å^2^)

**Table:**

	*x*	*y*	*z*	*U*_iso_*/*U*_eq_	Occ. (<1)
S1	0.64100 (9)	0.15466 (4)	0.63757 (5)	0.04732 (14)	
S2	0.47667 (10)	−0.01817 (4)	0.80937 (6)	0.05463 (16)	
C1	0.775 (3)	0.0231 (13)	0.6391 (16)	0.063 (3)	0.541 (13)
H1A	0.8165	0.0099	0.5476	0.076*	0.541 (13)
H1B	0.9197	0.0230	0.7097	0.076*	0.541 (13)
C2	0.6217 (17)	−0.0684 (4)	0.6704 (9)	0.0580 (18)	0.541 (13)
H2A	0.7152	−0.1339	0.7017	0.070*	0.541 (13)
H2B	0.5059	−0.0874	0.5866	0.070*	0.541 (13)
C1'	0.742 (3)	0.0127 (13)	0.6019 (19)	0.064 (4)	0.459 (13)
H1'A	0.6365	−0.0202	0.5217	0.076*	0.459 (13)
H1'B	0.9000	0.0149	0.5818	0.076*	0.459 (13)
C2'	0.7381 (17)	−0.0516 (5)	0.7321 (11)	0.057 (2)	0.459 (13)
H2'A	0.8802	−0.0357	0.8011	0.069*	0.459 (13)
H2'B	0.7374	−0.1303	0.7104	0.069*	0.459 (13)
C3	0.4657 (3)	0.12007 (16)	0.75830 (19)	0.0385 (4)	
C4	0.3248 (3)	0.19662 (16)	0.8105 (2)	0.0384 (4)	
C5	0.1840 (3)	0.16281 (18)	0.91489 (18)	0.0419 (4)	
C6	0.0463 (4)	0.24818 (18)	0.9638 (2)	0.0495 (5)	
H6	−0.0410	0.2314	1.0328	0.059*	
C7	0.0409 (3)	0.35039 (17)	0.9126 (2)	0.0477 (5)	
H7	−0.0534	0.4027	0.9462	0.057*	
C8	0.3154 (3)	0.31026 (17)	0.7579 (2)	0.0422 (4)	
C9	0.1463 (3)	0.49614 (15)	0.7608 (2)	0.0402 (4)	
C10	−0.0659 (3)	0.53405 (19)	0.6854 (2)	0.0517 (5)	
H10	−0.1967	0.4874	0.6672	0.062*	
C11	−0.0823 (4)	0.6429 (2)	0.6368 (2)	0.0613 (6)	
H11	−0.2248	0.6693	0.5854	0.074*	
C12	0.1097 (5)	0.71177 (19)	0.6640 (3)	0.0646 (6)	
H12	0.0981	0.7844	0.6301	0.078*	
C13	0.3191 (4)	0.67360 (19)	0.7411 (3)	0.0604 (6)	
H13	0.4486	0.7209	0.7608	0.072*	
C14	0.3393 (3)	0.56575 (17)	0.7898 (2)	0.0494 (5)	
H14	0.4820	0.5401	0.8419	0.059*	
N1	0.1675 (3)	0.38340 (13)	0.81222 (16)	0.0422 (4)	
O1	0.4251 (3)	0.34287 (12)	0.66800 (17)	0.0611 (4)	
O2	0.1866 (2)	0.06549 (12)	0.95739 (17)	0.0562 (4)	

### Atomic displacement parameters (Å^2^)

**Table:**

	*U*^11^	*U*^22^	*U*^33^	*U*^12^	*U*^13^	*U*^23^
S1	0.0552 (3)	0.0452 (3)	0.0482 (3)	0.0055 (2)	0.0264 (2)	0.0024 (2)
S2	0.0693 (3)	0.0355 (2)	0.0669 (3)	0.0066 (2)	0.0325 (3)	0.0060 (3)
C1	0.071 (4)	0.066 (6)	0.064 (7)	0.005 (3)	0.039 (4)	−0.022 (4)
C2	0.067 (4)	0.040 (2)	0.074 (4)	0.004 (2)	0.030 (3)	−0.007 (2)
C1'	0.111 (10)	0.034 (4)	0.059 (8)	0.014 (4)	0.048 (6)	−0.012 (4)
C2'	0.059 (4)	0.035 (3)	0.081 (5)	0.005 (3)	0.022 (4)	0.001 (3)
C3	0.0437 (10)	0.0379 (10)	0.0358 (9)	−0.0007 (8)	0.0123 (7)	0.0000 (7)
C4	0.0437 (9)	0.0369 (9)	0.0381 (9)	−0.0002 (7)	0.0167 (7)	0.0008 (8)
C5	0.0482 (10)	0.0415 (10)	0.0387 (9)	−0.0055 (9)	0.0154 (7)	0.0024 (9)
C6	0.0597 (12)	0.0503 (12)	0.0469 (11)	0.0006 (10)	0.0313 (9)	0.0002 (9)
C7	0.0556 (11)	0.0459 (12)	0.0484 (11)	0.0022 (9)	0.0272 (9)	−0.0053 (9)
C8	0.0501 (11)	0.0399 (10)	0.0412 (10)	0.0042 (8)	0.0199 (8)	0.0025 (8)
C9	0.0458 (9)	0.0376 (10)	0.0417 (9)	0.0042 (8)	0.0194 (7)	0.0006 (8)
C10	0.0436 (10)	0.0543 (12)	0.0589 (13)	0.0057 (9)	0.0144 (9)	−0.0045 (10)
C11	0.0611 (12)	0.0638 (15)	0.0593 (12)	0.0260 (12)	0.0127 (10)	0.0049 (12)
C12	0.0907 (17)	0.0430 (11)	0.0712 (14)	0.0188 (12)	0.0433 (13)	0.0104 (11)
C13	0.0627 (13)	0.0433 (12)	0.0831 (15)	−0.0024 (10)	0.0341 (11)	−0.0004 (11)
C14	0.0463 (10)	0.0441 (11)	0.0600 (14)	0.0013 (9)	0.0153 (9)	0.0000 (9)
N1	0.0508 (9)	0.0347 (8)	0.0465 (9)	0.0038 (7)	0.0227 (7)	−0.0003 (7)
O1	0.0833 (10)	0.0434 (8)	0.0717 (10)	0.0140 (8)	0.0525 (8)	0.0151 (7)
O2	0.0678 (9)	0.0442 (8)	0.0644 (9)	−0.0033 (7)	0.0323 (7)	0.0112 (7)

### Geometric parameters (Å, °)

**Table:**

S1—C3	1.732 (2)	C5—C6	1.432 (3)
S1—C1	1.761 (15)	C6—C7	1.323 (3)
S1—C1'	1.857 (16)	C6—H6	0.9300
S2—C3	1.732 (2)	C7—N1	1.377 (2)
S2—C2	1.813 (5)	C7—H7	0.9300
S2—C2'	1.850 (6)	C8—O1	1.231 (2)
C1—C2	1.479 (19)	C8—N1	1.396 (2)
C1—H1A	0.9700	C9—C10	1.376 (3)
C1—H1B	0.9700	C9—C14	1.379 (3)
C2—H2A	0.9700	C9—N1	1.441 (2)
C2—H2B	0.9700	C10—C11	1.388 (3)
C1'—C2'	1.48 (2)	C10—H10	0.9300
C1'—H1'A	0.9700	C11—C12	1.367 (4)
C1'—H1'B	0.9700	C11—H11	0.9300
C2'—H2'A	0.9700	C12—C13	1.370 (3)
C2'—H2'B	0.9700	C12—H12	0.9300
C3—C4	1.385 (3)	C13—C14	1.377 (3)
C4—C8	1.455 (3)	C13—H13	0.9300
C4—C5	1.466 (2)	C14—H14	0.9300
C5—O2	1.240 (2)		
			
C3—S1—C1	95.2 (5)	C8—C4—C5	120.70 (16)
C3—S1—C1'	98.4 (7)	O2—C5—C6	122.80 (17)
C1—S1—C1'	12.1 (9)	O2—C5—C4	121.01 (18)
C3—S2—C2	96.19 (18)	C6—C5—C4	116.20 (18)
C3—S2—C2'	94.75 (19)	C7—C6—C5	121.31 (18)
C2—S2—C2'	26.22 (19)	C7—C6—H6	119.3
C2—C1—S1	113.1 (9)	C5—C6—H6	119.3
C2—C1—H1A	109.0	C6—C7—N1	123.46 (17)
S1—C1—H1A	109.0	C6—C7—H7	118.3
C2—C1—H1B	109.0	N1—C7—H7	118.3
S1—C1—H1B	109.0	O1—C8—N1	119.78 (18)
H1A—C1—H1B	107.8	O1—C8—C4	123.65 (17)
C1—C2—S2	106.3 (7)	N1—C8—C4	116.55 (16)
C1—C2—H2A	110.5	C10—C9—C14	120.52 (19)
S2—C2—H2A	110.5	C10—C9—N1	120.09 (17)
C1—C2—H2B	110.5	C14—C9—N1	119.38 (17)
S2—C2—H2B	110.5	C9—C10—C11	119.1 (2)
H2A—C2—H2B	108.7	C9—C10—H10	120.4
C2'—C1'—S1	105.6 (11)	C11—C10—H10	120.4
C2'—C1'—H1'A	110.6	C12—C11—C10	120.5 (2)
S1—C1'—H1'A	110.6	C12—C11—H11	119.8
C2'—C1'—H1'B	110.6	C10—C11—H11	119.8
S1—C1'—H1'B	110.6	C11—C12—C13	119.9 (2)
H1'A—C1'—H1'B	108.7	C11—C12—H12	120.1
C1'—C2'—S2	111.9 (9)	C13—C12—H12	120.1
C1'—C2'—H2'A	109.2	C12—C13—C14	120.6 (2)
S2—C2'—H2'A	109.2	C12—C13—H13	119.7
C1'—C2'—H2'B	109.2	C14—C13—H13	119.7
S2—C2'—H2'B	109.2	C13—C14—C9	119.4 (2)
H2'A—C2'—H2'B	107.9	C13—C14—H14	120.3
C4—C3—S1	122.87 (14)	C9—C14—H14	120.3
C4—C3—S2	121.84 (15)	C7—N1—C8	121.74 (16)
S1—C3—S2	115.28 (11)	C7—N1—C9	119.40 (15)
C3—C4—C8	118.94 (16)	C8—N1—C9	118.86 (15)
C3—C4—C5	120.35 (18)		
			
C3—S1—C1—C2	29.1 (11)	O2—C5—C6—C7	−177.6 (2)
C1'—S1—C1—C2	−77 (5)	C4—C5—C6—C7	2.6 (3)
S1—C1—C2—S2	−40.8 (12)	C5—C6—C7—N1	−1.2 (3)
C3—S2—C2—C1	31.6 (9)	C3—C4—C8—O1	0.5 (3)
C2'—S2—C2—C1	−56.5 (9)	C5—C4—C8—O1	−178.25 (19)
C3—S1—C1'—C2'	−28.1 (12)	C3—C4—C8—N1	178.91 (17)
C1—S1—C1'—C2'	47 (5)	C5—C4—C8—N1	0.1 (3)
S1—C1'—C2'—S2	40.1 (13)	C14—C9—C10—C11	1.1 (3)
C3—S2—C2'—C1'	−33.7 (10)	N1—C9—C10—C11	−179.94 (18)
C2—S2—C2'—C1'	60.7 (11)	C9—C10—C11—C12	−0.3 (3)
C1—S1—C3—C4	175.0 (5)	C10—C11—C12—C13	−0.8 (4)
C1'—S1—C3—C4	−173.3 (6)	C11—C12—C13—C14	1.0 (4)
C1—S1—C3—S2	−5.4 (5)	C12—C13—C14—C9	−0.3 (3)
C1'—S1—C3—S2	6.3 (6)	C10—C9—C14—C13	−0.8 (3)
C2—S2—C3—C4	165.9 (4)	N1—C9—C14—C13	−179.81 (18)
C2'—S2—C3—C4	−167.8 (4)	C6—C7—N1—C8	−0.9 (3)
C2—S2—C3—S1	−13.8 (4)	C6—C7—N1—C9	178.73 (18)
C2'—S2—C3—S1	12.5 (4)	O1—C8—N1—C7	179.79 (18)
S1—C3—C4—C8	2.5 (3)	C4—C8—N1—C7	1.4 (3)
S2—C3—C4—C8	−177.09 (14)	O1—C8—N1—C9	0.2 (3)
S1—C3—C4—C5	−178.70 (14)	C4—C8—N1—C9	−178.25 (16)
S2—C3—C4—C5	1.7 (2)	C10—C9—N1—C7	−65.0 (2)
C3—C4—C5—O2	−0.6 (3)	C14—C9—N1—C7	114.0 (2)
C8—C4—C5—O2	178.16 (18)	C10—C9—N1—C8	114.7 (2)
C3—C4—C5—C6	179.24 (18)	C14—C9—N1—C8	−66.3 (2)
C8—C4—C5—C6	−2.0 (3)		

### Hydrogen-bond geometry (Å, °)

**Table:**

*D*—H···*A*	*D*—H	H···*A*	*D*···*A*	*D*—H···*A*
C1'—H1'A···O1^i^	0.97	2.43	3.300 (17)	148
C2—H2B···O1^i^	0.97	2.69	3.388 (8)	129
C7—H7···O2^ii^	0.93	2.36	3.259 (3)	163
C14—H14···O2^iii^	0.93	2.46	3.293 (3)	149
C11—H11···S1^iv^	0.93	2.90	3.756 (3)	153

Symmetry codes: (i) −*x*+1, *y*−1/2, −*z*+1; (ii) −*x*, *y*+1/2, −*z*+2; (iii) −*x*+1, *y*+1/2, −*z*+2; (iv) −*x*, *y*+1/2, −*z*+1.
